# Regulation of Intestinal Barrier Function and Gut Microbiota by Hot Melt Extrusion-Drug Delivery System-Prepared Mulberry Anthocyanin in an Inflammatory Bowel Disease Model

**DOI:** 10.3390/ph18040475

**Published:** 2025-03-27

**Authors:** Eun-Ji Go, Byeong Ryeol Ryu, Gyeong Ju Gim, Ye Rim Shin, Min Ji Kang, Min Jun Kim, Jong-Suep Baek, Jung Dae Lim

**Affiliations:** 1 Department of Bio-Health Convergence, Kangwon National University, Chuncheon 24341, Republic of Korea; a01040363654@kangwon.ac.kr (E.-J.G.); byeongryeol.ryu@csupueblo.edu (B.R.R.); tlsdpfladldi@kangwon.ac.kr (Y.R.S.); mzk1227@kangwon.ac.kr (M.J.K.); alswns1172@kangwon.ac.kr (M.J.K.); jsbaek@kangwon.ac.kr (J.-S.B.); 2 Institute of Cannabis Research, Colorado State University-Pueblo, 2200 Bonforte Blvd, Pueblo, CO 81001-4901, USA; 3 National Agrobiodiversity Center, National Academy of Agricultural Science, Rural Development Administration, Jeonju 54874, Republic of Korea; gyeongju159@korea.kr; 4 Department of Bio-Functional Material, Kangwon National University, Samcheok 25949, Republic of Korea

**Keywords:** mulberry, anthocyanin, hot-melt extrusion, drug release, gut microbiota, intestinal microbial flora, intestinal barrier, bioavailability, inflammatory disease

## Abstract

**Background/Objectives:** Anthocyanins (ACNs) derived from mulberry (*Morus alba* L.) exhibit potent antioxidant and anti-inflammatory activities. However, their low stability and bioavailability in physiological environments limit their therapeutic potential. This study aimed to enhance the stability and controlled release ACNs using a hot-melt extrusion drug delivery system (HME-DDS) formulation, HME-MUL-F2, and evaluate its effects on gut barrier function and microbiota composition in a DSS-induced colitis model. **Methods:** The anthocyanin content of HME-MUL-F2 was quantified and compared with that of raw mulberry extract. The formulation’s protective effects were assessed in Caco-2 and RAW 264.7 cells, confirming its biocompatibility and anti-inflammatory properties. The therapeutic efficacy was further evaluated in a dextran sulfate sodium (DSS)-induced inflammatory bowel disease (IBD) model, focusing on gut barrier integrity, inflammatory cytokine modulation, and gut microbiota composition. **Results:** HME-MUL-F2 significantly improved gut barrier function by upregulating tight junction proteins and reducing inflammatory cytokine levels in the colitis model. Moreover, the formulation modulated gut microbiota composition, promoting beneficial bacteria while suppressing pathogenic strains. HME-MUL-F2 administration led to a significant increase in the Bacteroidetes-to-Firmicutes ratio, which is associated with improved gut health. These results indicate that HME-MUL-F2 significantly enhances anthocyanin bioavailability, leading to improved gut health and potential therapeutic applications for inflammatory conditions. **Conclusions:** This study highlights the potential of HME technology for improving the stability, bioavailability, and therapeutic efficacy of anthocyanins. HME-MUL-F2 is a sustained-release formulation that enhances gut barrier function and modulates intestinal microbial balance in a DSS-induced inflammatory bowel disease model. These findings strongly suggest that the observed therapeutic effects of HME-MUL-F2 are primarily due to enhanced anthocyanin bioavailability and targeted delivery to the colon, although further clinical studies will provide more definitive confirmation.

## 1. Introduction

The gastrointestinal tract serves as a primary defense mechanism, where epithelial cells create a physical barrier to minimize direct contact with external substances [1]. These cells regulate the entry and exit of substances, as well as absorption and secretion [2]. The intestinal wall provides the first line of defense for the mucosa, protecting it from stomach and pancreatic secretions and bacterial antigens [3].

Intestinal barrier homeostasis depends on the relationship between the microbiome and the epithelium. The innate immune system, especially pattern recognition receptors in enterocytes, is essential for maintaining this homeostasis [4]. The intestinal barrier, including the microbiota, epithelial cells, and mucosal immunity, forms a defense unit interconnected through cytokines, antimicrobial peptides (AMPs), and metabolites [5]. Maintaining this barrier is crucial for the body’s anti-infective defense [6]. A damaged epithelium allows for the movement of various bacteria and toxins, leading to severe complications like sepsis and spontaneous bacterial peritonitis [7].

The intestinal epithelium recognizes microbe-associated molecular patterns (MAMPs) from microbial species, triggering inflammatory signals [8]. MAMPs, including lipopolysaccharides (LPS) in Gram-negative bacteria, lipoteichoic acid (LTA) in Gram-positive bacteria, and peptidoglycan, activate the NF-κB signaling pathway, induce pro-inflammatory interleukins, and maintain a controlled inflammatory state [9].

Enteric pathogens contribute to inflammatory bowel disease (IBD) by altering the intestinal barrier and microbiota, increasing inflammation, and releasing toxins [10]. The microbiota, consisting of millions of microorganisms, support metabolic, immune, and anti-infective processes [11]. Dysbiosis, a disruption in microbial balance, weakens the intestinal barrier, leading to infections and IBD [12].

Inflammatory bowel disease (IBD), encompassing Crohn’s disease and ulcerative colitis, is a chronic inflammatory condition of the gastrointestinal tract. Recent studies have highlighted a significant association between gut microbiota dysbiosis and the pathogenesis of IBD. Specifically, IBD patients exhibit reduced microbial diversity, with a decrease in beneficial bacteria such as Lachnospiraceae and Bifidobacterium species, and an increase in harmful bacteria like Proteobacteria and Fusobacterium species [13].

There is no permanent cure for IBD, and lifelong medication is often required. Maintaining remission and preventing inflammation recurrence are the main treatment goals [14]. Amino salicylates like 5-amino salicylic acid (5-ASA) are the preferred initial treatment for mild to moderate IBD symptoms, exerting anti-inflammatory effects through antioxidant activity [15]. However, rapid absorption from the small intestine reduces its availability in the inflamed colon, leading to side effects [16].

Mulberry (*Morus alba*), commonly known as white mulberry, is a fruit-bearing tree widely cultivated for its leaves, which are the primary food source for silkworms. The fruit, known as mulberry, is rich in various nutrients and bioactive compounds, including vitamins, minerals, dietary fibers, and phenolic compounds, particularly anthocyanins (ACNs) [17]. These compounds are responsible for the fruit’s vibrant color and have significant health benefits, such as antioxidant, anti-inflammatory, and anti-cancer properties [18]. Mulberry supports cardiovascular health, helps regulate blood sugar levels, enhances immune function, and shows potential for protecting against neurodegenerative diseases and improving digestive health due to its high fiber content and beneficial effects on gut microbiota [19].

Anthocyanins (ACNs) possess antioxidant, anti-inflammatory, and anticancer properties but display low bioavailability due to their instability [20]. In vivo studies show that ACN is absorbed most efficiently in the jejunum, with much less absorption in the duodenum [21]. Consequently, ACN’s impact on maintaining the balance of intestinal microbial flora in the colon is limited [22]. The antioxidant properties of anthocyanins are crucial in maintaining the balance of the gut microbiota and supporting the integrity of the intestinal barrier by reducing oxidative stress and inflammation [23].

Hot-melt extrusion (HME) technology enhances the bioavailability of poorly soluble substances [24]. HME is used in the pharmaceutical and food industries to increase the water solubility, particle stability, and antioxidant effects of compounds. The HME-MUL-F2 formulation, developed in the study “Hot-Melt Extrusion Enhances Antioxidant Effects of Mulberry on Probiotics and Pathogenic Microorganisms”, demonstrated the increased solubility and stability of ACNs. This formulation helps maintain the mucosal barrier by preserving the balance of intestinal microbiota and preventing the proliferation of harmful bacteria [25]. By using HME-DDS, ACN is safely delivered to the colon, without decomposing in stomach acid, improving ulcerative colitis caused by dextran sulfate sodium (DSS) by enhancing intestinal barrier function and controlling microorganisms.

Mulberry was chosen for this study due to its high content of bioactive compounds, particularly anthocyanins, which have been shown to have significant health benefits. The use of mulberry in combination with HME technology aims to overcome the bioavailability issues associated with anthocyanins and to enhance their therapeutic effects on intestinal health and inflammation. Compared to conventional anthocyanin formulations, HME-MUL-F2 enhances stability, scalability, and targeted colonic delivery, making it a promising alternative to existing delivery systems.

## 2. Results and Discussion

### 2.1. Toxicity of HME-MUL-F2 to Caco-2 and RAW 264.7 Cells

**Figure 1:**
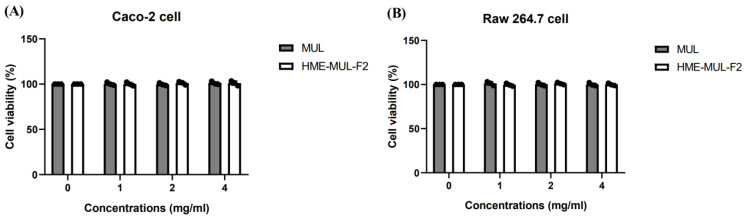
Effects of HME-DDS formulate (HME-MUL-F2) on cell viability in Caco-2 and RAW 264.7 cells. Increasing concentrations of mulberry raw materials (MUL) and HME-DDS formulate (HME-MUL-F2) in amounts of 1, 2, 4 mg/mL were applied to (**A**) Caco-2 cells and (**B**) RAW 264.7 cells. The cytotoxicity of RAW 264.7 cells and HME-MUL-F2 cells after 24 h was evaluated using the MTT assay. One-way ANOVA was conducted, followed by Duncan’s multiple range test, to analyze statistical differences at a 5% significance level (*p* < 0.05, *n* = 3).

To assess the toxicity of MUL and HME-MUL-F2 to Caco-2 and RAW 264.7 cells, an MTT assay was conducted. When the cells reached 80% confluence, they were pretreated with MUL and HME-MUL-F2 at concentrations of 1, 2, and 4 mg/mL for 24 h. Studies have been conducted to evaluate the cytotoxicity of extracts containing anthocyanins such as C3G (cyanidin-3-glucoside) and C3R (cyanidin-3-rutinoside) in Caco-2 and RAW 264.7 cells. However, no studies have been identified that assess the toxicity of these compounds at high concentrations exceeding 0.1 mg/mL [26]. Cell viability was measured and found to reach 100% across all treatment groups, with no significant difference compared to the results for the untreated group. This indicates that MUL and HME-MUL-F2 did not exhibit toxicity, even at high doses. Therefore, a concentration of 2 mg/mL, which showed efficacy in previous intestinal microbiome improvement experiments, was used for further studies. There was no statistically significant difference in cell viability (Figure 1, *p* < 0.05).

Additionally, the anthocyanin contents of the mulberry extracts used in this study are presented in Table 1. Table 1 shows the contents of C3G and C3R of mulberry extracts, comparing raw materials (MUL) and the HME-MUL-F2 formulation, and the anthocyanin content of MUL was determined to be 43.13 ± 2.63 mg/g DW, which is comparable to previously reported values for mulberry (*Morus alba*) fruit ranging from 4.11 to 52.88 mg/g DW of C3G mL [27]. On the other hand, HME-MUL-F2 exhibited an anthocyanin content of 325.02 ± 11.12 mg/g DW, which is significantly higher than that of raw mulberry extract. HME-MUL-F2 exhibited significantly higher concentrations of anthocyanins compared to raw mulberry (*p* < 0.05), indicating enhanced stability and bioavailability through the hot-melt extrusion (HME) process.

### 2.2. Effect of HME-MUL-F2 on Tight Junction Integrity of Caco-2 Cells

Cellular models are more accessible than in vivo models and offer a method to investigate substance effects without risking animal welfare [29]. Caco-2 cells, derived from human colorectal adenocarcinoma, are widely used as an intestinal barrier model due to their strong correlation with passive permeability [30]. These cells form a polarized monolayer characteristic of human enterocytes [31]. However, they cannot produce mucus, so RAW 264.7 cells are used to better mimic the internal environment [32]. An in vitro model was developed using Caco-2 cells on the apical side and RAW 264.7 cells on the basolateral side of a Transwell insert [33]. This setup allows for the investigation of transport, metabolism, and cell–cell interactions relevant to gut inflammation [33]. LPS stimulation of RAW 264.7 cells increased cytokine secretion and reduced TEER value [34], indicating immune stress and damage to tight junctions [35].

LPS treatment in the basolateral compartment caused pro-inflammatory cytokine secretion, affecting Caco-2 cells and leading to barrier function failure. TEER decreased from 840 Ω × cm^2^ to 696 Ω × cm^2^ over 48 h. The effect of HME-MUL-F2 and MUL on Caco-2 cell tight junctions was evaluated. After pretreatment with MUL-F2 at 2 mg/mL for 24 h, the TEER value was measured, while treating the basolateral compartment with LPS at 2 µg/mL for 48 h [36].

**Figure 2:**
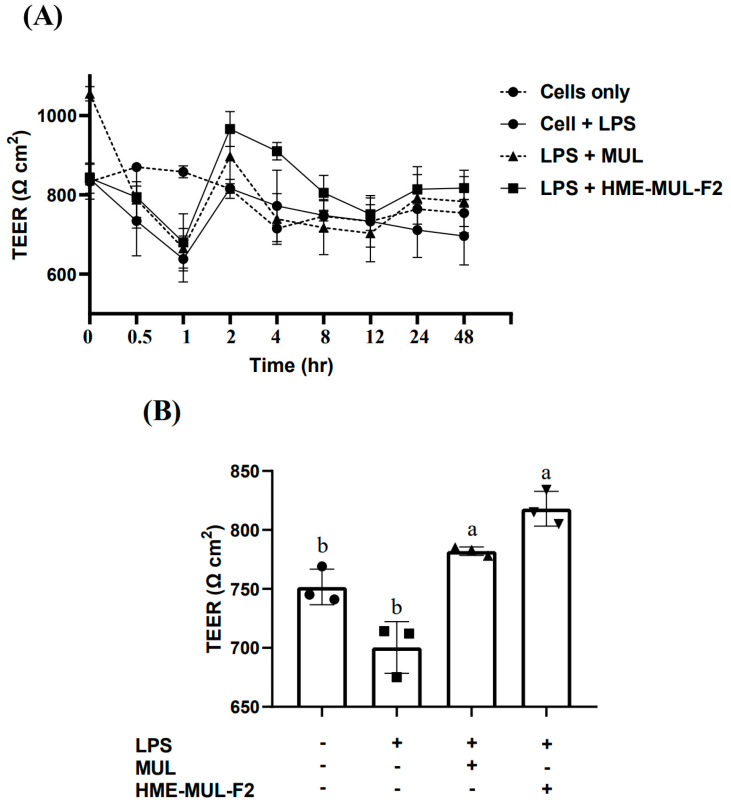
Preventive effects of HME-DDS formulate (HME-MUL-F2) on reduced tight junction integrity by treatment with LPS in an in vitro gut inflammation model. Tight junction integrity of Caco-2 cells (apical compartment) in the absence or presence of mulberry raw materials (MUL) and HME-DDS formulate (HME-MUL-F2) by treatment with 2 μg/mL LPS in RAW 264.7 cells (basolateral compartment) was determined by (**A**) time course and (**B**) final (treatment with LPS after 48 h) TEER values. Different letters (a,b) of error bars within each column indicate significant differences among treatments. One-way ANOVA was conducted, followed by Duncan’s multiple range test, to analyze the statistical differences at a 5% significance level (*p* < 0.05, *n* = 3).

LPS treatment alone decreased TEER to 696 Ω × cm^2^, 0.92 times lower than that for the untreated group (754 Ω × cm^2^). However, with 24 h pretreatment of MUL and HME-MUL-F2, followed by LPS treatment, TEER values were 783 Ω × cm^2^ and 817 Ω × cm^2^, respectively (Figure 2A, *p* < 0.05). Through their intrinsic self-repair mechanisms, Caco-2 cells possess inherent self-repair capabilities that can restore tight junction integrity over time [37]. TEER measurements demonstrated that HME-MUL-F2 effectively mitigated LPS-induced increases in permeability in Caco-2 monolayers, suggesting its protective role in regards to intestinal barrier integrity. The recovery of TEER values within 24 h and their further elevation beyond control levels at 48 h indicate accelerated barrier repair, potentially due to the sustained release of anthocyanins (Figure 2B). Previous studies have reported that anthocyanins from aronia berries similarly prevent LPS-induced disruption of Caco-2 barrier function. Additionally, Jiang et al. have shown that barrier function restoration is a time-dependent process, supporting our observation that HME-MUL-F2 promotes faster TEER recovery [38].

The higher TEER value for HME-MUL-F2 compared to MUL is likely due to the structure of sugar molecules and macromolecules in HME-MUL-F2, which may enhance ACN uptake in Caco-2 cells. However, final TEER values at 48 h were not significantly different between MUL and HME-MUL-F2. These results align with reports that mulberry anthocyanins (ACN) reduce tight junction permeability to LPS [39], helping to maintain the intestinal barrier.

The hot-melt extrusion (HME) process has been shown to improve the stability and solubility of anthocyanins, leading to a sustained release profile [28]. This enhancement ensures a more consistent and prolonged interaction with the intestinal epithelium, thereby supporting tight junction integrity. Moreover, the enhanced performance of HME-MUL-F2 may be attributed to the HME process, which improves anthocyanin solubility and stability, leading to sustained release and prolonged retention in the intestinal environment [28]. Unlike non-extruded formulations, which exhibit rapid anthocyanin degradation within 180 min, HME-MUL-F2 maintained significant anthocyanin levels for extended durations [28]. These findings indicate that the optimized formulation facilitates gradual release, resulting in superior intestinal barrier protection compared to that of raw mulberry materials (MUL).

### 2.3. Effect of HME-MUL-F2 on Inhibition of Cytokine Production

**Figure 3:**
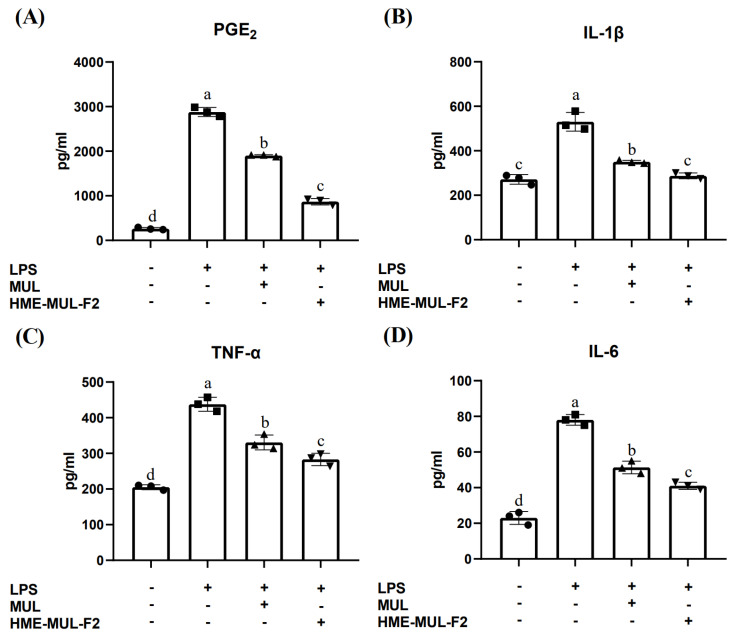
Impact of HME-DDS formulation (HME-MUL-F2) on proinflammatory cytokine production in Caco-2 cells. Caco-2 monolayers were pre-treated apically with 2 mg/mL of mulberry raw materials (MUL) and HME-DDS formulation (HME-MUL-F2) for 24 h. This was followed by incubation, with RAW 264.7 cells exposed to lipopolysaccharide (LPS) in the basolateral compartment for an additional 24 h. ELISA kit analysis for cytokine production of (**A**) prostaglandin E_2_ (PGE_2_), (**B**) interleukin-beta (IL-1β), (**C**) tumor necrosis factor-α (TNF-α), and (**D**) IL-6 was performed. Different letters (a–d) of error bars within each column indicate significant differences among treatments. One-way ANOVA was conducted, followed by Duncan’s multiple range test, to analyze statistical differences at a 5% significance level (*p* < 0.05, *n* = 3).

LPS treatment in the Caco-2 monolayer reduced TEER levels, indicating compromised tight junctions. Treatment with MUL and HME-MUL-F2 prevented this reduction and decreased inflammatory cytokine expression in the apical compartment. PGE_2_ levels increased significantly with LPS treatment (262.00 ± 25.94 pg/mL to 2879.33 ± 102.10 pg/mL). MUL and HME-MUL-F2 pretreatment reduced PGE_2_ levels to 1902.67 ± 20.50 pg/mL, although not to untreated levels (Figure 3A, *p* < 0.05). IL-1β increased from 271.33 ± 21.78 pg/mL to 530.33 ± 42.15 pg/mL with LPS. MUL and HME-MUL-F2 reduced IL-1β to 350.00 ± 7.21 pg/mL and 287.00 ± 13.00 pg/mL, respectively, with HME-MUL-F2 showing a statistically significant reduction to near untreated levels (Figure 3B, *p* < 0.05). TNF-α levels increased to 437.67 ± 19.50 pg/mL with LPS. MUL and HME-MUL-F2 reduced TNF-α to 330.67 ± 20.82 pg/mL and 278.67 ± 17.47 pg/mL, respectively, although not to untreated levels (205.00 ± 7.00 pg/mL, *p* < 0.05). IL-6 increased from 23.00 ± 3.61 pg/mL to 78.00 ± 3.00 pg/mL with LPS but was reduced to 51.33 ± 3.51 pg/mL with MUL and 41.00 ± 2.00 pg/mL with HME-MUL-F2 treatment (Figure 3C,D, *p* < 0.05).

Gene expression and production of inflammatory cytokines like IFN-α, TNF-α, IL-1β, IL-6, IL-17, and IL-22 form a complex network that can lead to chronic inflammation and autoimmune diseases when imbalanced [40]. LPS can cause chronic inflammation of the intestinal mucosal barrier, but MUL and HME-MUL-F2 can inhibit this by reducing inflammatory cytokines. HME-MUL-F2 significantly reduced the levels of pro-inflammatory cytokines (PGE_2_, TNF-α, IL-1β, and IL-6) in LPS-stimulated Caco-2/RAW264.7 co-culture, demonstrating its anti-inflammatory potential. Previous studies have shown that anthocyanins from natural sources inhibit the nuclear translocation of NF-κB p65 and prevent IκB-α degradation, leading to reduced cytokine production [41]. In our study, the downregulation of PGE_2_, TNF-α, IL-1β, and IL-6 further supports the hypothesis that HME-MUL-F2 modulates NF-κB signaling, leading to a suppressed inflammatory response. Additionally, alginate—an excipient in the HME-MUL-F2 formulation—has been reported to exhibit independent anti-inflammatory effects by modulating macrophage activation and cytokine secretion [42].

The potential synergistic interaction between anthocyanins and alginate may further contribute to the anti-inflammatory effects observed in our study. HME-MUL-F2, which includes alginate and ascorbyl palmitate and is derived using extrusion technology, improves intestinal barrier function more effectively than does the raw material alone. It can be used as a preventive and therapeutic agent to maintain intestinal mucosal integrity.

Following LPS treatment, a decrease in TEER values was observed, followed by a gradual recovery. This phenomenon is consistent with findings from other studies, such as the work of Jiang et al. [38]. Despite cellular damage induced by LPS, the cells can recover relatively quickly due to their intrinsic self-repair mechanisms. Therefore, the initial decrease and subsequent increase in TEER values after LPS treatment can be interpreted as a reflection of the processes of cellular damage and recovery.

These results suggest that HME-MUL-F2 may exert its anti-inflammatory effects by suppressing pro-inflammatory cytokine expression.

### 2.4. Effects of HME-MUL-F2 on Gene Expression Related to Tight Junction

**Figure 4:**
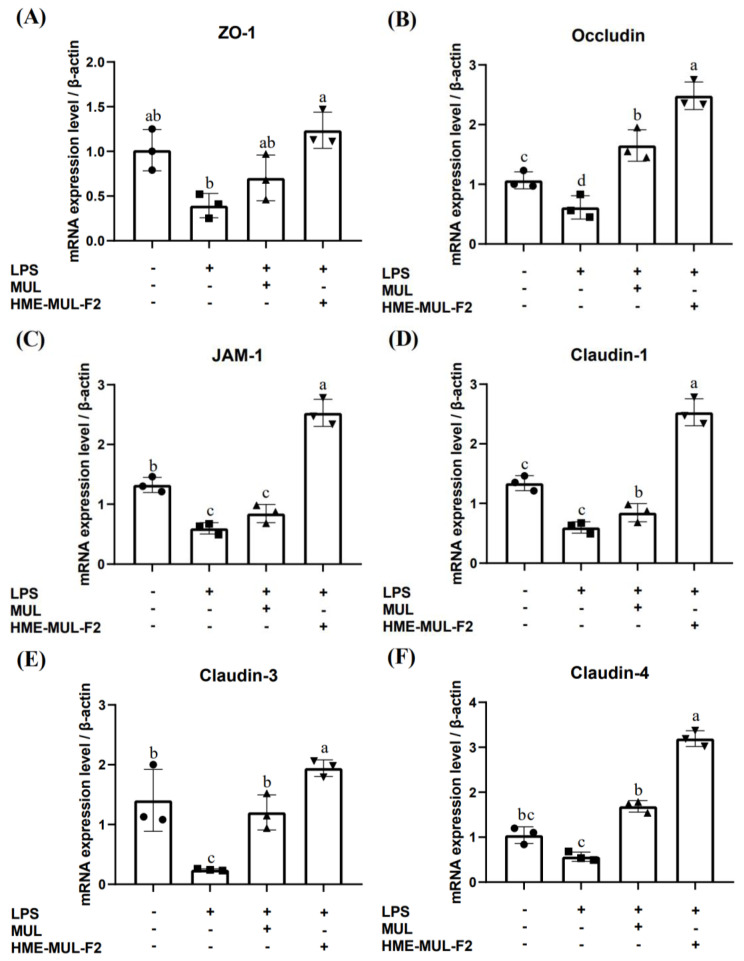
The comparison of mRNA gene expressions of the tight junction obtained from HME-DDS formulate (HME-MUL-F2) in Caco-2 cells. Quantitative RT-PCR analysis was conducted to examine the gene expression levels of tight junction proteins, including (**A**) ZO-1, (**B**) occludin, (**C**) JAM-1, (**D**) claudin-1, (**E**) claudin-3, and (**F**) claudin-4. LPS: lipopolysaccharide, MUL: mulberry raw materials, HME-MUL-F2: HME-DDS formulation using mulberry. Different letters (a–d) of error bars within each column indicate significant differences among treatments. One-way ANOVA was conducted, followed by Duncan’s multiple range test, to analyze statistical differences at a 5% significance level (*p* < 0.05, *n* = 3).

LPS was used in the basolateral compartment to induce an inflammatory reaction in RAW 264.7 cells. Tight junction protein gene expression in Caco-2 cells was measured to evaluate the protective effects of HME-MUL-F2 on intestinal barrier function (Figure 4). LPS treatment reduced mRNA levels of the tight junction (TJ)-related genes ZO-1 (0.39), occludin (0.61), JAM-1 (0.60), claudin-1 (0.35), claudin-3 (0.24), and claudin-4 (0.57) compared to the results for the control (A relative value, assuming the control group is set as 1 for comparison, *p* < 0.05).

These results align with reports that LPS-induced inflammation disrupts the small intestine epithelial barrier by altering TJ proteins [43]. MUL treatment increased occludin, claudin-1, and claudin-4 mRNA levels above control levels, while HME-MUL-F2 increased all TJ proteins above control levels, even after LPS treatment.

HME-MUL-F2 significantly boosted the expression of genes reduced by LPS, demonstrating twice the effectiveness of MUL. Tight junctions are crucial barriers against harmful substances like bacteria and toxins [44]. Endothelial TJs regulate vascular permeability and leukocyte leakage [45], and their dysfunction can lead to continuous inflammation and tissue damage [46]. Inflammation rearranges and modifies transcription in epithelial and endothelial cells, increasing permeability [47]. Natural substances can mitigate TJ rearrangement and help control inflammation-induced permeability [48]. ACN supplementation reduces intestinal permeability and endotoxemia [49].

HME-DDS promotes TJ-related gene expression more effectively than that of the untreated groups, likely due to excipients in HME-MUL-F2. Sodium alginate enhances the immune response and strengthens intestinal cell membranes [50]; poloxamer 188 regulates cytokines and increases occludin and ZO-1 proteins [51].

### 2.5. HME-MUL-F2 Ameliorates DSS-Induced Colitis

**Figure 5:**
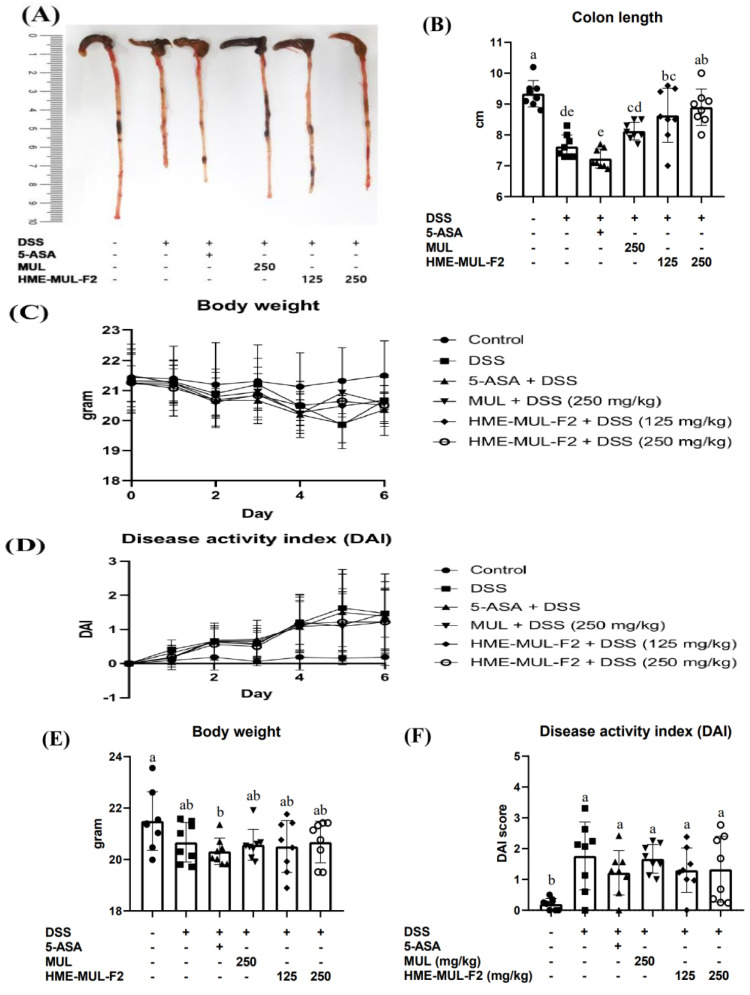
Effects of HME-MUL-F2 and MUL on histopathological parameters. (**A**) Picture of colon; (**B**) colon length; (**C**,**E**) body weight; (**D**,**F**) disease activity index. DSS: dextran sulfate sodium; 5-ASA: 5-aminosalicylic acid; MUL: mulberry raw materials; HME-MUL-F2: HME-DDS formulation using mulberry. Different letters (a–e) of error bars within each column indicate significant differences among treatments. One-way ANOVA was conducted, followed by Duncan’s multiple range test to analyze statistical differences at a 5% significance level (*p* < 0.05, *n* = 8).

To confirm the preventive effects of MUL and HME-MUL-F2 on DSS-induced colitis in mice, treatments included 5-ASA (150 mg/kg), MUL (250 mg/kg), and HME-MUL-F2 (125 mg/kg, 250 mg/kg), alongside DSS (250 mg/kg) for 7 days. Colon length, a marker of inflammation, was significantly reduced in the DSS group (9.55 ± 0.47 cm to 7.75 ± 0.48 cm). MUL and HME-MUL-F2 treatments resulted in longer colon lengths, i.e., MUL + DSS (8.10 ± 0.27 cm), HME-MUL-F2 + DSS (125 mg/kg: 8.53 ± 0.41 cm, 250 mg/kg: 8.85 ± 0.72 cm), indicating protection against colon shortening (Figure 5A,B, *p* < 0.05).

Weight change, an indicator of disease severity, and the disease activity index (DAI) were evaluated. MUL and HME-MUL-F2 treatments resulted in weight and DAI scores similar to those of the untreated group, except for the 5-ASA positive control. Although not highly significant, weight gain and reduced DAI scores were observed compared to the results for the DSS group, showing protective potential (Figure 5C–F).

HME-MUL-F2 treatment significantly mitigated weight loss and improved disease activity index (DAI) in DSS-induced colitis mice. These effects align with previous findings on mulberry anthocyanins, where supplementation restored tight junction protein expression (ZO-1, occludin, claudin-3) and alleviated DSS-induced colitis symptoms [52].

Notably, our study demonstrated that HME-MUL-F2 achieved comparable or superior protective effects at half the dosage of the raw mulberry extract (MUL), suggesting that the optimized formulation enhances therapeutic efficacy. This is consistent with studies on sustained-release formulations of 5-ASA, where improved drug delivery resulted in enhanced anti-inflammatory outcomes [53]. Thus, HME-MUL-F2 can improve DSS-induced colitis.

### 2.6. The Effects of HME-MUL-F2 on Serum Inflammatory Cytokines in DSS-Induced Colitis

IL-1β is a key cytokine in immune and inflammatory responses, secreted by monocytes, mast cells, smooth muscle cells, and endothelial cells. It activates lymphocytes and promotes immune cell infiltration at injury sites, with higher expression in UC patients than in healthy individuals [54]. TNF-α enhances phagocytosis and cytokine secretion and influences the NF-κB pathway, critical in both inflammation and IBD [55]. Both TNF-α and IL-1 upregulate MCP-1, impacting various immune cells, and are elevated in the mucosal tissues of CD and UC patients and in experimental colitis models [56].

IBD diagnosis and management are often invasive and costly. Biomarkers like MPO offer a reliable method to monitor IBD activity, indicating disease onset and severity [57]. MPO, found in the neutrophils, indicates neutrophil infiltration and inflammation, with studies showing increased MPO in DSS-induced colitis, suggesting MCP-1’s role in colitis pathogenesis [58].

**Figure 6:**
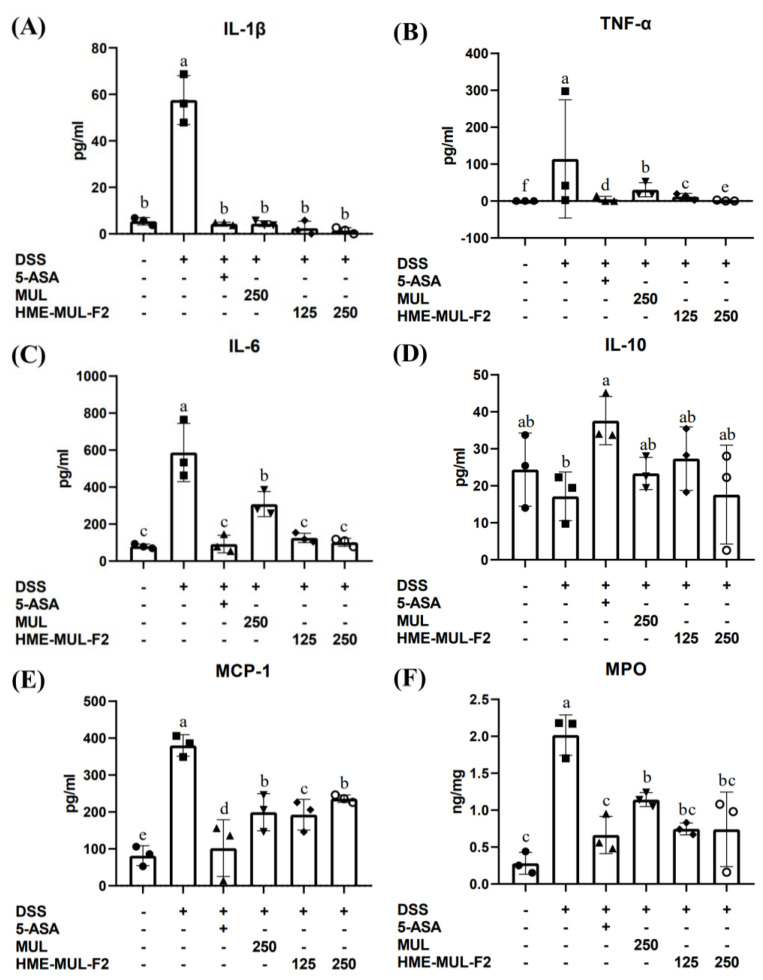
The effect of MUL and HME-MUL-F2 on inflammatory cytokines and MPO. (**A**) IL-1β; (**B**) TNF-α; (**C**) IL-6; (**D**) IL-10; (**E**) MCP-1; (**F**) MPO. DSS: dextran sulfate sodium; 5-ASA: 5-aminosalicylic acid; MUL: mulberry raw materials; HME-MUL-F2: HME-DDS formulation using mulberry. Different letters (a–f) of error bars within each column indicate significant differences among treatments. One-way ANOVA was conducted, followed by Duncan’s multiple range test to analyze statistical differences at a 5% significance level (*p* < 0.05, *n* = 3).

MUL and HME-MUL-F2 reduced the DSS-induced expression of IL-1β, TNF-α, IL-6, and MCP-1, while increasing IL-10 expression (Figure 6, *p* < 0.05). HME-MUL-F2, containing alginate and ascorbyl palmitate and utilizing HME technology, improves intestinal inflammation, even in small amounts [59].

### 2.7. Histopathology of DSS Induced Colitis

**Figure 7:**
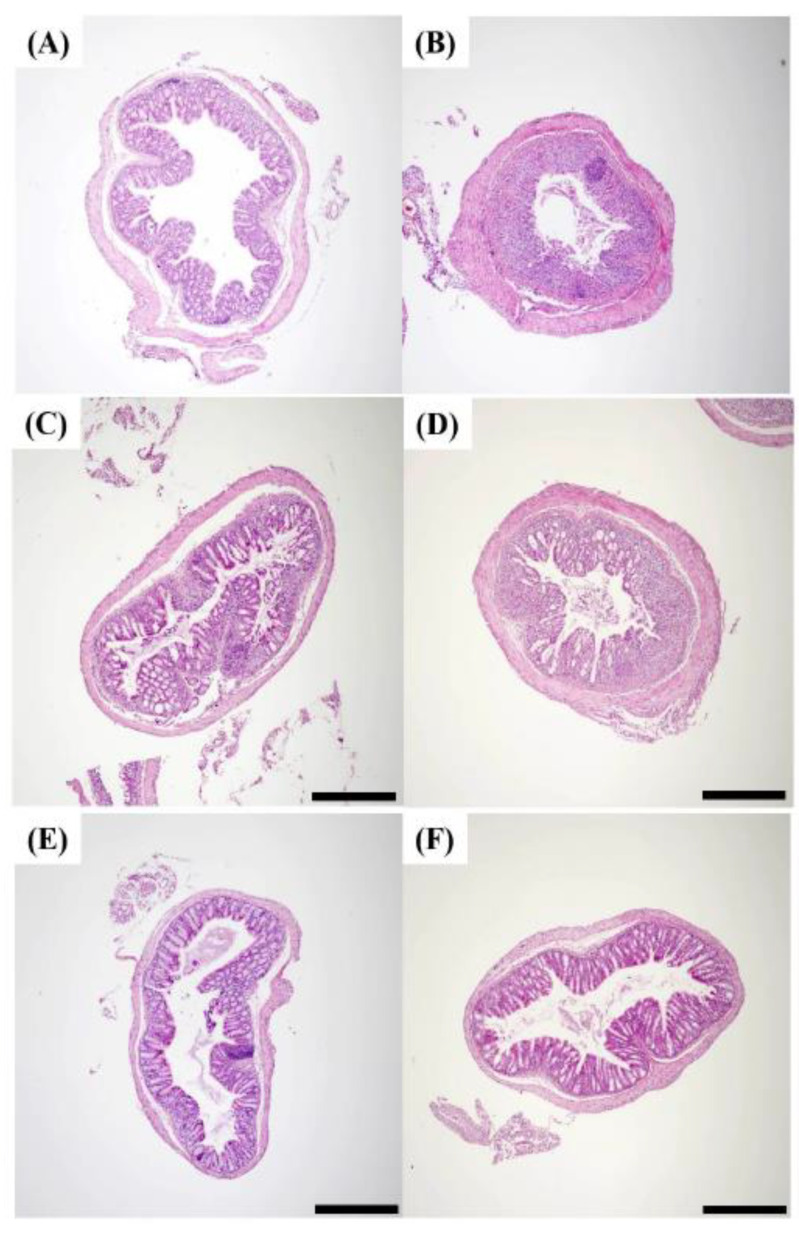
Hematoxylin and eosin (H&E) staining of colon tissues (arrows, ×40; scale bar, 500 μm). (**A**) Control; (**B**) dextran sulfate sodium (DSS); (**C**) DSS + 5-aminosalicylic acid (5-ASA); (**D**) DSS + mulberry raw material (MUL), 250 mg/kg; (**E**) DSS + HME-DDS formulation using mulberry (HME-MUL-F2), 125 mg/kg; (**F**) DSS + HME-MUL-F2, 250 mg/kg.

**Figure 8:**
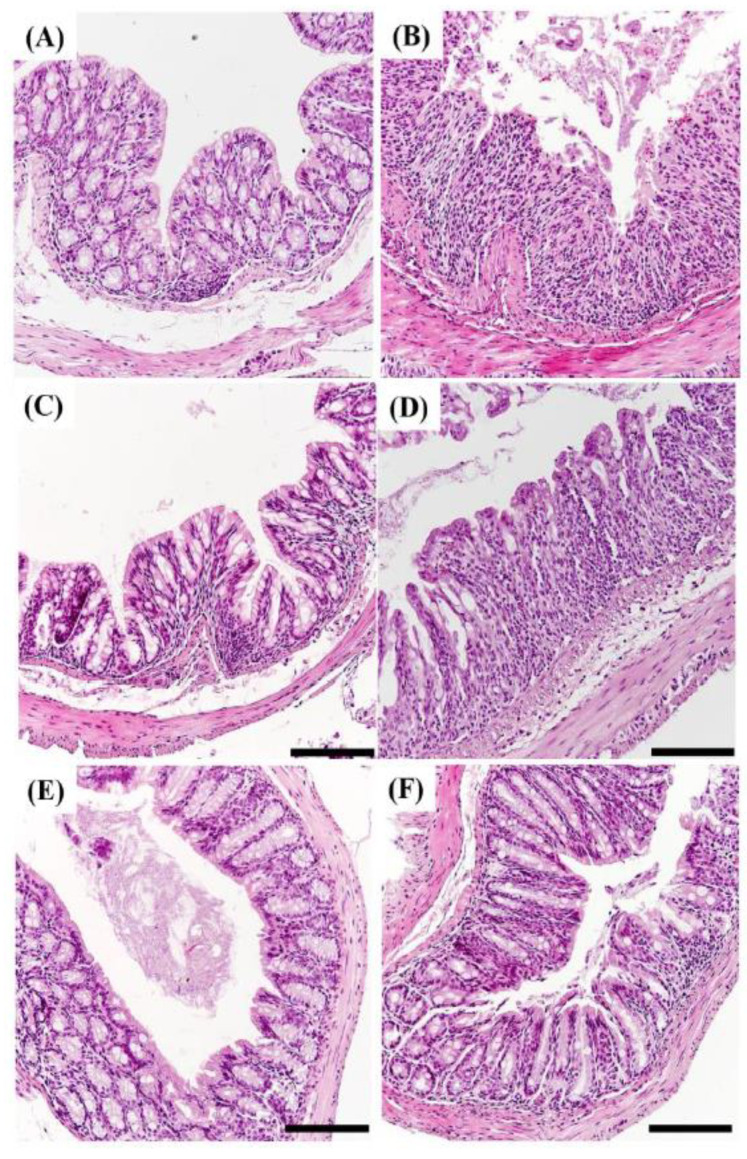
Hematoxylin and eosin (H&E) staining of colon tissues (arrows, ×200; scale bar, 100 μm). (**A**) Control; (**B**) dextran sulfate sodium (DSS); (**C**) DSS + 5-aminosalicylic acid (5-ASA); (**D**) DSS + mulberry raw material (MUL), 250 mg/kg; (**E**) DSS + HME-DDS formulation using mulberry (HME-MUL-F2), 125 mg/kg; (**F**) DSS + HME-MUL-F2, 250 mg/kg.

A hematoxylin and eosin stain assay (H&E staining) was performed to determine whether morphological changes appeared in the epithelium of the mouse small intestine. Hematoxylin stains the portion corresponding to the nucleus of the tissue in purple, and eosin stains the part corresponding to the cytoplasm of the tissue in red. When inflammation occurs in the intestine, inflammatory cells corresponding to macrophages and T cells infiltrate the lower part of the villus, which is called immune cell infiltration. As a result of H&E staining, it was confirmed that inflammatory cell infiltration appeared clearly in the small intestine of DSS group mice compared to the results for the control group (Figure 7 and Figure 8). This result is consistent with the report that inflammatory cell infiltration occurs in the large intestine of mice administered with DSS [60].

These results show that DSS induces an inflammatory response in both the large and small intestines of mice. Colonic sections from control mice showed intact mucosa, with no signs of mucosal inflammation. It was confirmed that the epithelium was not damaged, there was no edema in the mucosa, and neutrophils and macrophages did not infiltrate into the mucosa and submucosa. In contrast, colonic sections from mice with colitis showed severe histologic signs. Observation revealed epithelial damage, mucosal edema, and the infiltration of neutrophils and macrophages into both the mucosal and submucosal layers [61].

In the HME-MUL-F2-treated group, no mucosal edema was observed, and the infiltration of neutrophils and macrophages was significantly reduced, leading to an inflammatory cell infiltration rate comparable to that of the healthy control group. The extent of epithelial reconstitution and tissue recovery in the HME-MUL-F2-treated group was comparable to that observed in the healthy control mice, indicating effective mucosal healing. These results indicate that HME-MUL-F2 treatment was associated with improved histological outcomes compared to the results for MUL. This suggests a potential role of enhanced anthocyanin bioavailability; further studies are needed to directly establish whether this effect is due to increased anthocyanin delivery to the colon.

### 2.8. HME-MUL-F2 Alleviated DSS-Induced Colonic Barrier Dysfunction

One common symptom of IBD is the loss of tight junctions (TJs), leading to epithelial breakdown and altered TJ protein composition. These changes contribute to diarrhea and increase the permeability of the intestinal barrier, allowing for pathogen penetration and inflammation [62]. TJs are essential for maintaining intestinal barrier integrity and selective permeability. In IBD, TJ loss is a predictor for diagnosing Crohn’s disease and preventing disease recurrence [63]. Tight junctions consist of proteins such as zonula occludens (ZO), occludin, claudins, and junctional adhesion molecules (JAMs) [64].

Patients with IBD exhibit heightened intestinal permeability due to defective TJs. Claudin-1 and claudin-4 enhance the epithelial barrier, while occludin regulates paracellular permeability [65]. ZO-1 interacts with β-actin and myosin II to construct the cytoskeleton [66]. In Crohn’s disease (CD), there is a decrease in occludin, claudin-3, claudin-5, claudin-8, and JAM-A, but an increase in claudin-2 [67]. In ulcerative colitis (UC), occludin, claudin-1, claudin-4, and JAM-A are decreased, while claudin-2 is increased [68].

**Figure 9:**
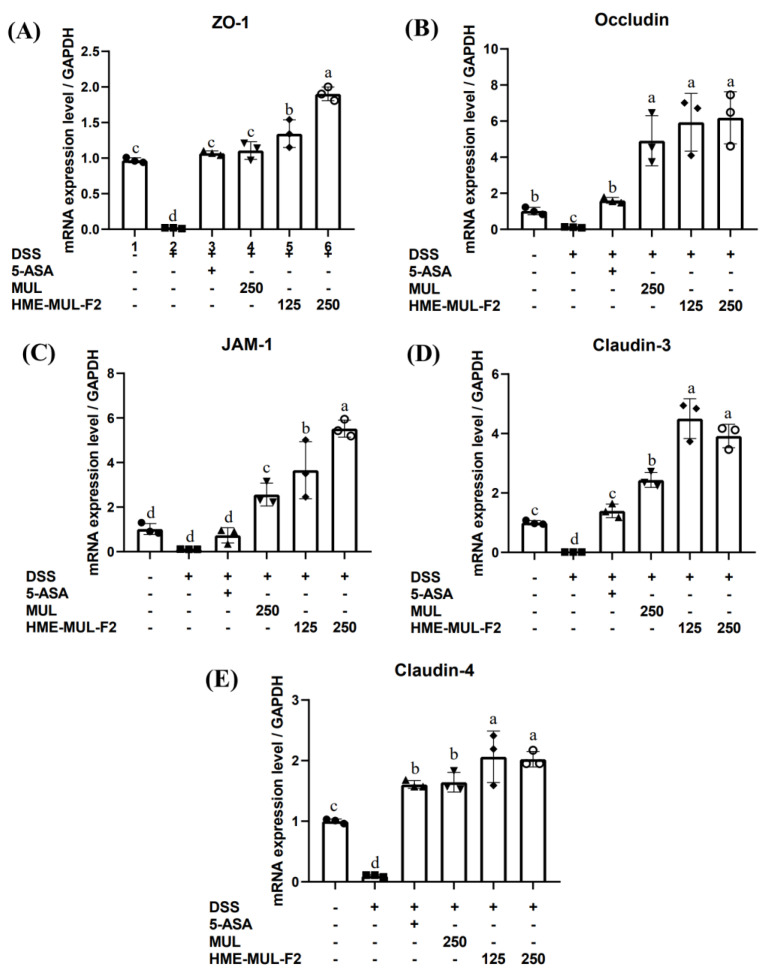
Effect of MUL and HME-MUL-F2 on intestinal barrier protection. (**A**) ZO-1; (**B**) occludin; (**C**) JAM-1; (**D**) claudin-3; (**E**) claudin-4. DSS: dextran sulfate sodium; 5-ASA: 5-aminosalicylic acid; MUL: mulberry raw material; HME-MUL-F2: HME-DDS formulation using mulberry. Different letters (a–d) of error bars within each column indicate significant differences among treatments. One-way ANOVA was conducted, followed by Duncan’s multiple range test to analyze statistical differences at a 5% significance level (*p* < 0.05, *n* = 3).

The effect of MUL and HME-MUL-F2 on mRNA expression of TJ-related proteins (ZO-1, occludin, JAM-1, claudin-3, claudin-4) in DSS-induced colitis was evaluated using RT-qPCR. After DSS treatment, mRNA levels of TJ-related genes were significantly reduced, i.e., ZO-1 (0.02), occludin (0.12), JAM-1 (0.11), claudin-3 (0.01), and claudin-4 (0.10) compared to the results for the control group (Figure 9, *p* < 0.05). These results are consistent with reports that cytokines in DSS-induced inflammation loosen the epithelial barriers by altering protein expression and movement [69].

After 5-ASA treatment, the expression of TJ proteins returned to control levels. MUL and HME-MUL-F2 treatment resulted in statistically higher TJ-related gene expression levels than those of the control. HME-MUL-F2 (125 mg/kg) showed higher gene expression than did MUL (250 mg/kg), indicating that HME-DDS has a significant effect on intestinal inflammatory modulators in DSS-induced colitis and can improve intestinal damage.

### 2.9. HME-MUL-F2 Suppressed the Expression of p-NF-κB p65 and IκB in DSS-Induced Colitis

Inflammatory responses are mediated by pro-inflammatory cytokines like TNF, IL-1β, and IL-6 through the activation of NF-κB, a key inflammatory transcription factor. NF-κB consists of five proteins: p50, p52, c-Rel, RelA (p65), and RelB [70]. Normally, NF-κB proteins are maintained in the cytosol by binding with IκB proteins. Upon stimulation, the p65-p50 dimer of NF-κB is released from IκB through phosphorylation, leading to ubiquitylation and the proteasomal degradation of IκB [71]. The liberated p65-p50 dimer translocates to the nucleus and induces gene expression involved in the inflammatory response [72].

The inhibition of NF-κB activation by HME-MUL-F2 may be attributed to the stabilization of anthocyanins, which have been reported to block the phosphorylation of IκBα and reduce the nuclear translocation of NF-κB p65.

Clinical studies suggest that NF-κB p65 oligonucleotides significantly reduce NF-κB p65 and cytokine expression in the mucosa, improving clinical symptoms [73] NF-κB, a heterodimer of p50 and p65, is regulated by IκB and IKK. Inactive IKK is bound to IκB in the cytoplasm. Noxious stimuli activate IKK, leading to the phosphorylation of IκBα, which activates NF-κB, causing it to move to the nucleus and bind to DNA, encoding inflammatory mediators [74].

**Figure 10:**
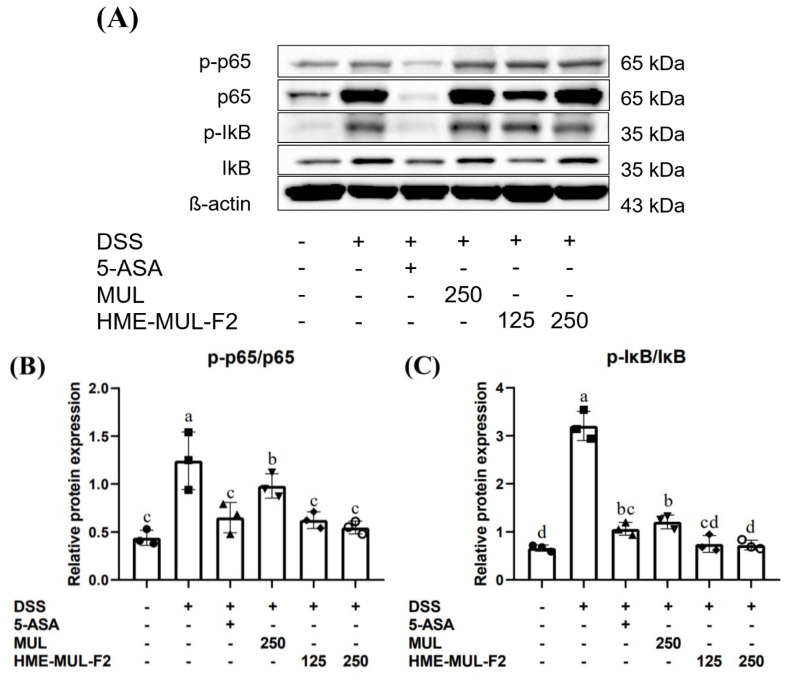
Effect of MUL and HME-MUL-F2 on the activation of NF-κB in the colon of DSS-induced colitis mice. Protein samples underwent Western blot analysis with specific antibodies; β-actin was used as an internal control. The results were consistent across three independent experiments, with one representative experiment shown. The expression levels of p-p65, p65, p-IκB, and IκB in colon tissue were assessed by Western blotting (**A**). Relative quantification of p-p65 and p65 protein expression was performed (**B**). The relative quantification of p-IκB and IκB protein expression (**C**). Different letters (a–d) of error bars within each column indicate significant differences among treatments. One-way ANOVA was conducted, followed by Duncan’s multiple range test to analyze statistical differences at a 5% significance level (*p* < 0.05, *n* = 3).

When bound to IκB, NF-κB does not function as a transcription factor. However, when IκB is phosphorylated by stimulants like DSS, it degrades, allowing NF-κB to enter the nucleus and promote the synthesis of inflammatory mediators [75]. In assessing the anti-inflammatory effect of HME-MUL-F2 on NF-κB expression (Figure 10, *p* < 0.05), it was found that DSS-induced NF-κB expression in the nucleus was reduced in a concentration-dependent manner by HME-MUL-F2 treatment. Additionally, the phosphorylated NF-κB p65 (p-p65) and phosphorylated IκB (p-IκB) levels increased with DSS treatment but were suppressed by HME-MUL-F2, indicating that its anti-inflammatory effect may be related to blocking cytokine-mediated NF-κB activation. These results suggest that HME-MUL-F2 may exert its anti-inflammatory effects by inhibiting the NF-κB signaling pathway, preventing the degradation of IκBα and thereby suppressing pro-inflammatory cytokine expression.

### 2.10. Effects of HME-MUL-F2 on Gut Microbiota

Recent studies have highlighted the role of gut microbiota in IBD. The human gut microbiome, consisting of over 1000 bacterial species, includes key phyla like Bacteroidetes, Firmicutes, Actinobacteria, and Proteobacteria [76]. This microbiome supports digestion, nutrient metabolism, and energy supply. Disruption of the microbial balance promotes inflammation and disrupts homeostasis [77]. Increased intestinal permeability in IBD can lead to bacterial translocation, exacerbating inflammation [78]. Microbial metabolites like short-chain fatty acids and bile acids help control inflammation [79]. Reduced microbial diversity is linked to IBD severity [80].

**Figure 11:**
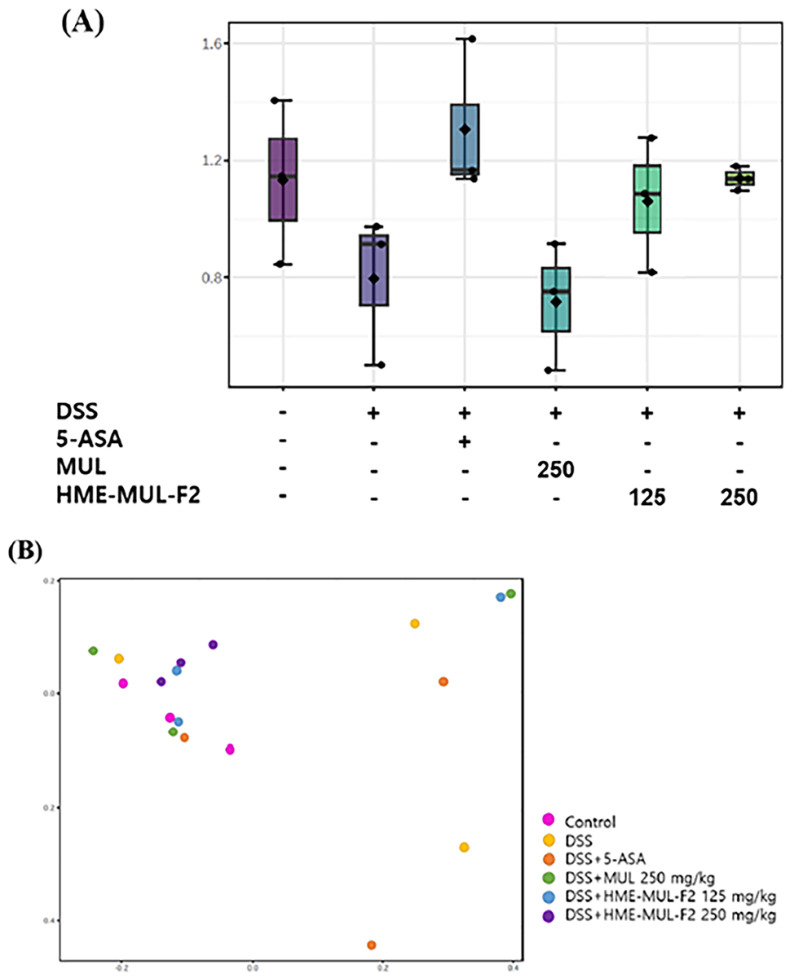
Metagenomic analysis of intestinal flora following dextran sulfate sodium (DSS), 5-aminosalicylic acid (5-ASA), mulberry raw material (MUL), and HME-DDS formulation using mulberry (HME-DDS-F2) treatments at the phylum level. (**A**) Shannon index; (**B**) PCoA plot.

**Figure 12:**
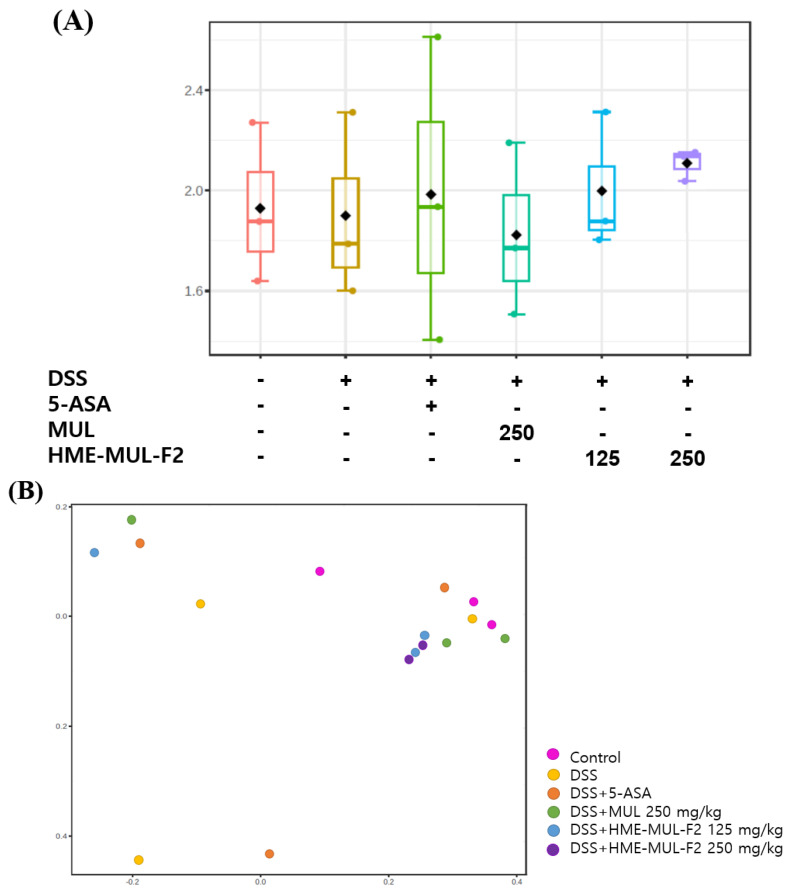
Metagenomic analysis of intestinal flora following dextran sulfate sodium (DSS), 5-aminosalicylic acid (5-ASA), mulberry raw material (MUL), and HME-DDS formulation using mulberry (HME-DDS-F2) treatments at the genus level. (**A**) Shannon index; (**B**) PCoA plot.

The Shannon diversity index and principal coordinates analysis (PCoA) were used to assess microbial diversity. Higher Shannon index values indicate greater diversity [81]. PCoA visualizes data similarity, showing that microbial imbalances are crucial in UC progression [82]. Alpha diversity decreased in DSS-induced colitis compared to the results for the controls (Figure 11A and Figure 12A). MUL (250 mg/kg) did not maintain alpha diversity, but HME-MUL-F2 did. Beta diversity showed a clustering of the control, HME-MUL-F2, and MUL groups, while the DSS group was distinct (Figure 11B and Figure 12B). HME-MUL-F2 maintains microbial composition and diversity in DSS treatment.

Several natural compounds and probiotic formulations have been investigated for their protective effects against DSS-induced colitis, including *Bacteroides ovatus* V975 (engineered to express TGF-β1), *Clostridium butyricum* MIYAIRI 588, and *Faecalibacterium prausnitzii* [83]. These formulations primarily exert their effects by modulating gut microbiota composition, promoting anti-inflammatory cytokines, or enhancing mucosal healing. In comparison, While the mechanisms differ, HME-MUL-F2 shows comparable or superior efficacy to those of other natural formulations by providing both anti-inflammatory and gut-protective benefits.

The enhanced effectiveness of HME-MUL-F2 compared to MUL may be attributed to the use of appropriate excipients in the hot-melt extrusion (HME) process, which facilitates the formation of a colloidal dispersion system. The enhanced gut microbiota modulation observed with HME-MUL-F2 may be partially due to the improved bioavailability of anthocyanins, allowing for greater interaction with intestinal microbiota and a subsequent reduction in inflammation. Unlike pH-sensitive coatings, which are highly depend on gastric transit variations, HME-MUL-F2 ensures consistent and prolonged release, allowing for more effective colonic targeting.

**Figure 13:**
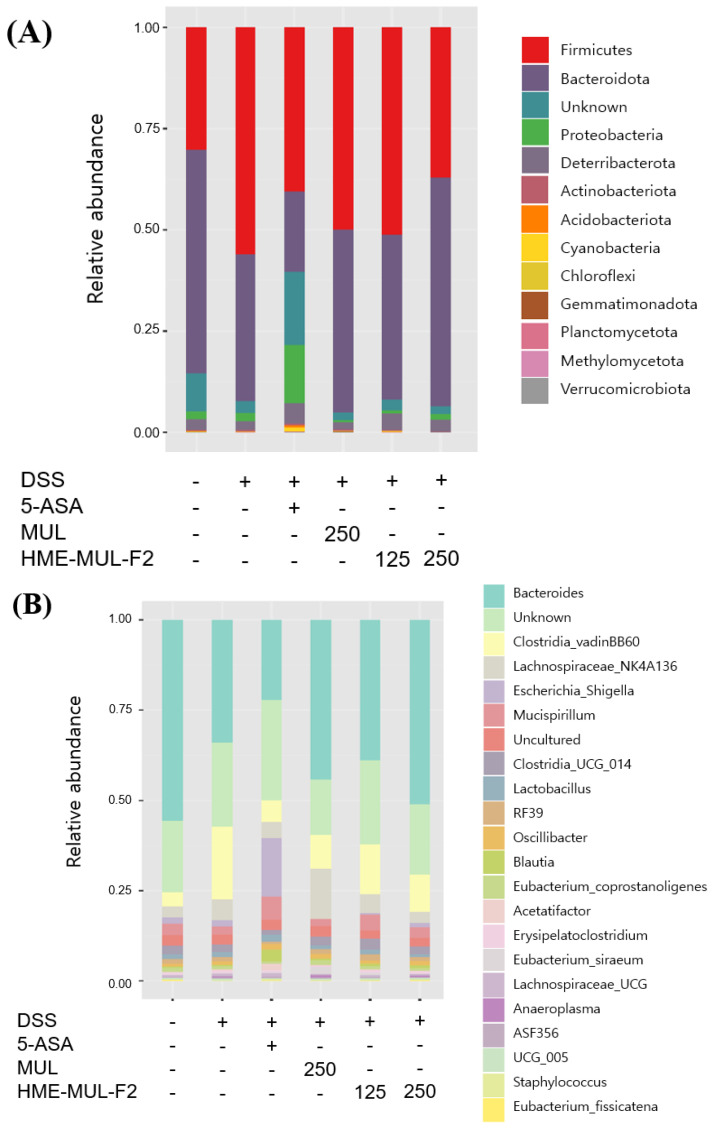
Metagenomic analysis of intestinal flora following dextran sulfate sodium (DSS), 5-aminosalicylic acid (5-ASA), mulberry raw material (MUL), and HME-DDS formulation using mulberry (HME-DDS-F2) treatments. (**A**) Phylum level; (**B**) genus level.

Colitis patients often exhibit a decreased Firmicutes/Bacteroides ratio, reduced biodiversity, and an imbalance in intestinal bacteria [84]. After DSS administration, Firmicutes, Bacteroidota, and Proteobacteria increased, indicating epithelial dysfunction [84]. DSS treatment increased Firmicutes and decreased Bacteroidota (Figure 13A). Following the 5-ASA treatment, both Firmicutes and Bacteroidota decreased. MUL and HME-MUL-F2 treatments increased Firmicutes and Bacteroidota.

At the genus level, colitis mice showed decreased Bacteroides and increased Lachnospiraceae_NK4A136 and Escherichia-Shigella [85]. MUL and HME-MUL-F2 treatments reversed this imbalance by increasing beneficial bacteria. Escherichia-Shigella is a pathogenic bacterium that promotes physiological changes and tissue infiltration in the intestinal epithelium [86]. Lachnospiraceae_NK4A136 correlates positively with colitis pathology [87]. MUL treatment showed high levels of Lachnospiraceae_NK4A136, but HME-MUL-F2 treatment reduced it, overcoming colitis.

HME-MUL-F2 treatment strengthened the barrier function by reducing harmful bacteria (Lachnospiraceae_NK4A136 and Escherichia-Shigella) and increasing beneficial bacteria (Bacteroides). These results align with reports that polymer nanoparticles relieve mucosal inflammation by protecting the mucosal layer and regulating macrophage function [88]. Therefore, HME-MUL-F2 can be used as a dietary component for preventing and treating colitis, suggesting its anti-ulcerative colitis effect by improving intestinal bacterial balance.

Recent advances in natural polymer-based drug delivery systems have significantly improved the bioavailability and therapeutic efficacy of bioactive compounds, particularly polyphenols such as anthocyanins. These natural biopolymer matrices offer enhanced stability, controlled release, and targeted delivery, making them promising candidates for gastrointestinal drug administration [89].

Dietary fibers and polysaccharides, including alginate, pectin, and chitosan, have been utilized as sustained-release carriers for polyphenols, allowing for prolonged retention and gradual absorption in the intestinal tract [90].

Traditional anthocyanin formulations, such as simple aqueous extracts or freeze-dried powders, often suffer from low stability and poor bioavailability due to rapid degradation in physiological conditions. In contrast, HME-MUL-F2 improves anthocyanin stability by incorporating polymeric excipients, which protect the bioactive compounds from oxidative and enzymatic degradation.

Various nano- and microencapsulation techniques have been developed to enhance the stability and intestinal absorption of polyphenols, including anthocyanins [91]. For instance, chitosan–pectin composite nanoparticles have been utilized as mucoadhesive carriers for anti-inflammatory compounds, improving their retention time in the gastrointestinal tract and enabling targeted release in the colon [92]. Similarly, liposomal and exosome-like nanoparticle formulations derived from plant sources have been shown to enhance polyphenol delivery and bioactivity [93].

Liposomal and nanoparticle-based delivery systems have been explored to enhance anthocyanin bioavailability; however, these systems often face challenges related to high production costs, limited scalability, and stability issues during storage. In comparison, HME-MUL-F2 offers a cost-effective and scalable alternative, utilizing hot-melt extrusion (HME) technology to create a solid dispersion system that facilitates sustained release and targeted delivery to the colon.

pH-sensitive or enteric-coated formulations are designed to bypass gastric degradation and enhance colonic delivery, but their efficiency is highly dependent on variations in individual gastric transit times and intestinal pH levels. In contrast, HME-MUL-F2 achieves sustained release through matrix-controlled dissolution, ensuring a more consistent and prolonged release profile, thereby enhancing local bioavailability at the site of action in the colon.

While conventional sustained-release systems often rely on hydrophilic polymers for controlled drug release, they may not effectively prevent early degradation in the upper gastrointestinal tract. HME-MUL-F2, by contrast, combines hydrophobic and mucoadhesive polymers, creating a protective matrix that enhances both stability and retention time, leading to improved therapeutic efficacy.

These findings indicate that natural polymer-based carriers not only enhance bioavailability but may also deliver additional biological benefits, such as gut microbiome modulation and immune regulation. Future studies could explore whether HME-MUL-F2 could be further optimized through its incorporation into such nanoformulations. The findings of this study suggest that HME-MUL-F2 could serve as a novel bioactive formulation with potential applications in inflammatory bowel disease (IBD) management. Compared to conventional anti-inflammatory agents such as 5-ASA, natural anthocyanin-based formulations offer the advantage of lower toxicity and enhanced gut microbiota compatibility [94].

Given the increasing interest in functional foods and nutraceuticals, HME-MUL-F2 could also be explored as a dietary supplement for gut health maintenance or as an adjunct therapy for patients with chronic inflammatory conditions. Moreover, incorporating biopolymer-based or nanoencapsulated formulations may further optimize its bioavailability and targeted release, paving the way for potential clinical trials.

Future research should focus on pharmacokinetic studies to confirm the absorption dynamics and sustained-release properties of HME-MUL-F2, along with gut microbiota analyses to determine its potential role in microbiome modulation. These advancements could provide a deeper understanding of how HME-MUL-F2 can be integrated into next-generation natural compound-based therapeutics.

## 3. Materials and Methods

### 3.1. Materials

The preparation of HME-MUL-F2 followed previously established methodologies. A detailed description of the formulation process, including the composition and extrusion parameters, is included in Ref. [28]. HME-MUL-F2 was prepared using a twin-screw extruder (STS-25HS, co-rotating intermeshing type, Pyeongtaek, Republic of Korea). The raw mulberry powder was mixed with excipients including ascorbyl palmitate (5%), mannitol (35%), sodium alginate (5%), and poloxamer 188 (5%) to enhance the stability and controlled release properties. The extrusion parameters were set as follows: feeding rate, 40 g/min; screw speed, 150 rpm; temperature, 70–100 °C. This process was adapted from our previous work [28] to improve the stability and bioavailability of anthocyanins.

In this study, the mulberry raw material (MUL) and HME-MUL-F2, which involves the use of excipients and the HME process on MUL, were utilized. The mulberries were collected from the Rural Development Administration in 2020, identified as *Morus alba* L., immediately washed, frozen, and stored at −20 °C until use. The HME-MUL-F2 formulation was able to delay the drug release rate in vitro, increase lactic acid bacteria, reduce harmful bacteria, and enhance antioxidant effects.

### 3.2. Cell Culture Conditions

To examine the effect of HME-MAL-F2 on tight junctions in the human small intestine, Caco-2 cells (30,037.1, Korean Cell Line Bank, Seoul, Republic of Korea) were placed in the apical compartment with a semi-permeable membrane, while RAW264.7 macrophages (40,071, Korean Cell Line Bank, Seoul, Republic of Korea) were co-cultured in the basolateral compartment. This in vitro gut inflammation model was used to study the transport, metabolism, and cell–cell interactions of the pharmacological components. The cell culture used penicillin–streptomycin solution, PBS, DMEM, 0.05% trypsin-EDTA solution, and FBS from Gibco, Grand Island, NY, USA, as well as 30 mm cell culture dishes from SPL Life Sciences [95]. Caco-2 cells were cultured in DMEM medium with 10% FBS, 1% antibiotics, and 5% CO_2_ at 37 °C [96].

### 3.3. Toxicity Evaluation

The impact of MUL and HME-MUL-F2 samples on cell stimulation and viability was assessed using the MTT test. RAW 264.7 cells (passage: 28), at 4 × 10^4^ cells/well, and Caco-2 cells (passage: 36), at 5 × 10^4^ cells/well, were incubated in a 96-well plate until 80% confluence was reached. The samples were diluted in culture medium to concentrations of 1, 2, and 4 mg/mL, pre-treated for one hour, and then treated for 24 h. After treatment, 10 µL of MTT solution (5 mg/mL) was added to each well and incubated for 4 h at 37 °C. The reaction was stopped by adding 200 µL of DMSO after removing the supernatant. The purple formazan was dissolved by gently shaking the plate in the dark. Subsequently, 50 µL from each well was transferred to a 96-well plate, and absorbance was measured at 570 nm using a microplate reader. Cell viability (%) was calculated using the following formula:

Cell viability (%) = [(A_tretment_ − A_blank_)/A_control_ − A_blank_] × 100

A: absorbance                                                                                    

### 3.4. In Vitro Inflammation Model

To study the effects of HME-MAL-F2 on tight junctions in the human small intestine, Caco-2 cells (passage: 36) were cultured in the apical compartment of a 6-well Transwell insert plate at a density of 1 × 10^5^ cells/well with 2 mL of medium [97]. The medium was changed every 2–3 days for 21 days. Care was taken during medium exchanges to avoid disturbing the membrane. In a 6-well plate, 1 × 10^5^ RAW 264.7 cells (passage: 28) were planted per well, with 3 mL of medium in the basolateral compartment, for 48 h.

After the culture period, the Transwell insert plate with Caco-2 cells was transferred to a 6-well plate containing RAW 264.7 cells. All media were replaced with serum-free DMEM [98].

### 3.5. TEER Measurement

Once Caco-2 cells formed an intact monolayer with tight junctions (TEER > 800 Ω × cm^2^); MAL and HME-MAL-F2 samples (2 mg/mL) were added to the apical wells and cultured for 24 h [99]. The basolateral wells were washed with PBS, and then 2 μg/mL of LPS in serum-free DMEM was added and cultured for another 24 h.

The integrity of tight junctions was verified by measuring TEER (transepithelial electrical resistance) using a Millicell ERS-2 resistance meter. The probe was positioned with the long side perpendicular to the bottom of the basolateral well and the short side slightly immersed in the medium of the apical well. Before measuring the next well, the probe was washed with PBS. TEER values were measured in triplicate and calculated using the following formula:

TEER (transepithelial electrical resistance) = resistance (Ω) × membrane area (cm^2^).

### 3.6. Cytokine and RNA Analysis

To assess the permeability increase and the resulting transfer of inflammatory cytokines from RAW 264.7 cells to the apical compartment, levels of NO, IL-1β, PGE2, and TNF-α were measured using Invitrogen ELISA kits. The cytokine content was analyzed in the apical compartment with Caco-2 cells to determine the effect of HME-MAL-F2 on maintaining tight junction integrity. If tight junctions were disrupted, cytokines from the basolateral RAW 264.7 cells would appear in the apical compartment.

For RNA analysis, Caco-2 cells treated with LPS were analyzed 24 h after treatment with MUL and HME-MUL-F2 samples. The cells were washed with 2 mL of PBS at 4 °C and collected. Total RNA was extracted using the Hybrid-R kit and dissolved in DEPC-H2O [100]. The RNA concentration was measured at 260/280 nm using a NanoDrop Spectrophotometer. Only RNA samples with an A260/A280 ratio of 1.8–2.1 were used. The isolated total RNA was stored at −70 °C.

### 3.7. Quantitative RT-PCR (qRT-PCR) Analysis

Total RNA was converted into cDNA using the Hyperscript 2X RT Master Mix. A 20 μL reaction mixture included the master mix, oligo dT, total RNA, and RNase-free water. cDNA synthesis was performed using a SimpliAmp Thermal Cycler with priming at 42 °C for 5 min, reverse transcription at 55 °C for 60 min, and inactivation at 95 °C for 5 min. Synthesized cDNA was stored at 4 °C for qRT-PCR.

Gene expression levels were measured using the Light Cycler 96 system, with specific primers. The 20 μL reaction mixture contained SYBR Green PCR master mix, forward and reverse primers, cDNA, and distilled water. PCR cycling involved 10 min at 95 °C for initial denaturation, followed by 40 cycles of 15 s at 95 °C, 30 s at 55 °C, and 30 s at 72 °C. The ΔCt value was calculated by subtracting β-actin’s Ct value from the target gene’s Ct value, and ΔΔCt was obtained by subtracting the control group’s average ΔCt from the experimental group’s ΔCt. Data were analyzed using the 2^−ΔΔCt^ method to analyze the relative changes in gene expression from real-time quantitative PCR experiments. Primer sequences for tight junction-related genes and β-actin were produced in a manner similar to that used by Kim et al. (2020) [101].

### 3.8. Animal Preparation

Animal studies were carried out with permission from Gyeongsang National University’s Animal Ethics Committee (approval number: GNU-230303-M0040, 3 March 2023). Male Balb/c mice (20 ± 2 g, 6 weeks old) were purchased from Orient Bio (Seongnam, Republic of Korea) and were specific pathogen-free (SPF). The mice were kept at a temperature of 23 ± 2 °C, 55–60% relative humidity, and a regular 12/12-h light/dark cycle. Following a week of acclimatization, each mouse was randomly assigned to one of six groups, with eight mice per group (*n* = 8). Oral administration was performed in each of the following six groups: the untreated group, the DSS group (250 mg/kg), the 5-aminosalicylic acid (5-ASA) group (150 mg/kg), the MUL group (250 mg/kg), and the two HME-MUL-F2 groups (125 mg/kg and 250 mg/kg). To induce colitis, the experimental groups were treated with a 2.5% DSS (MFCD00081551, MP Biomedical, Santa Ana, CA, USA) solution for 7 days.

### 3.9. Clinical Indicators and Pathological Evaluation

Clinical indicators, including body weight loss, stool consistency, and fecal blood, were monitored daily to assess disease activity. These parameters were used to calculate the disease activity index (DAI), following the methodology described by Khater et al. (2022) [102]. Weight loss was measured on a scale from 0 to 4 (0: no change, 1: 1–5% loss, 2: 5–10% loss, 3: 10–20% loss, 4: >20% loss). Stool viscosity, occult blood, and clinical scores were each rated from 0 to 4 (0: no change, 1: +, 2: ++, 3: +++, 4: ++++).

After euthanasia, colonic tissues were harvested for histopathological examination. Tissues were fixed in 10% neutral-buffered formalin, embedded in paraffin, sectioned at 5 μm, and stained with hematoxylin and eosin (H&E) for microscopic evaluation, as previously detailed by Khater et al. (2022) [102].

### 3.10. Cytokine, Chemokine, and Protein Analysis

Colon tissue was crushed using the BioMasher II device, and the supernatant was centrifuged at 4 °C and 12,000 rpm for 10 min. The enzyme solution was measured using the MPO expression ELISA kit (Thermo Fisher Scientific, Waltham, MA, USA). For serum cytokine measurement, blood was drawn from the mouse’s abdominal vena cava, centrifuged for 20 min at 4 °C at 3500 rpm, and the serum was separated. Cytokines IL-1β, IL-6, IL-10, MCP-1, and TNF-α were measured using kits from Thermo Fisher.

For protein analysis, colon tissue was lysed using RIPA buffer and BioMasher II, then centrifuged at 4 °C and 12,000 rpm for 30 min. Protein concentration was quantified using the Bradford method. Thirty micrograms of protein were electrophoresed on a 10% SDS-PAGE gel and transferred onto a PVDF membrane using an iBlot2 device. The membrane was blocked with 5% non-fat dried milk, incubated overnight at 4 °C with primary antibodies (for IκB, p65, p-IκB, β-actin; Cell Signaling, Danvers, MA, USA), washed with TBST, and then incubated for one hour with secondary antibodies at room temperature [92]. After washing, the membrane was treated with ECL solution (Intron, Seongnam, Republic of Korea). Protein bands were quantified using ChemiDoc XRS+ software Image Lab 6.1 (Bio-Rad, Hercules, CA, USA), and the results were analyzed as the ratio of relative expression to β-actin.

### 3.11. cDNA Synthesis and qPCR from Colon Tissue

A 1 cm section above the upper rectum was collected from the colon and stored at −80 °C immediately after collection. RNA was isolated using the RNeasy Mini Kit (Qiagen, Hilden, Germany), and its concentration and purity was assessed by measuring the absorbance at 260 and 280 nm. Only RNA samples with an OD260/280 ratio of 1.8 or higher were used for cDNA synthesis and qPCR.

cDNA was synthesized from total RNA using the TOP script cDNA synthesis kit (Enzynomics, Daejeon, Republic of Korea). qPCR was performed using MIC equipment (BMS, New York, NY, USA) with TOP real SYBR Green qPCR premix and specific primers. The qPCR protocol included initial denaturation at 95 °C for 10 min, followed by 50 cycles of 95 °C for 10 s, 60 °C for 15 s, and 72 °C for 15 s. GAPDH was used as the reference gene, and relative gene expression was calculated using the 2^−ΔΔCt^ method (Table 2).

### 3.12. Intestinal Microbial Community Metagenomic Analysis

Intestinal contents were collected from excised colon tissue, stored at a low temperature, and kept at −80 °C until DNA extraction. Feces from the colon were scraped and used for analysis. DNA was isolated using the FastDNA spin kit (MPBio, Santa Ana, CA, USA). After amplicon PCR with primers 518F (5′-CCAGCAGCCGCGGTAATACG-3′) and 805R (5′-GACTACCAGGGTATCTAATCC-3′), the PCR products were purified using Ampure XP. Indexing PCR was then performed with the Illumina Nextera XT index kit, followed by secondary purification. Sequencing was conducted via the iSeq 100 platform using a paired-end (151 bp × 2) method. The paired-end reads were merged and trimmed, based on quality criteria. The VSEARCH technique was used to identify operational taxonomic units (OTUs) at a 97% sequence identity threshold, and the RDP classifier was employed for taxonomic assignment. Alpha diversity and heatmap clustering analyses were performed using MicrobiomeAnalyst.

### 3.13. Statistical Analysis

The findings are shown as averages. The statistical analysis was conducted using SPSS software (version 16.0, IBM, Armonk, NY, USA). One-way ANOVA was performed, followed by Duncan’s multiple range test, to analyze statistical differences at a 5% significance level (*p* < 0.05, *n* = 3). Due to the small sample size, the results should be interpreted with caution.

## 4. Conclusions and Prospectives

This study developed HME-MUL-F2 using hot-melt extrusion (HME) technology to enhance the bioavailability and sustained release of C3G and C3R in mulberry. The optimized formulation was designed to protect active compounds, improve absorption, and enable controlled release, thereby potentially improving the stability and bioavailability of anthocyanins in the gastrointestinal environment.

Previous studies have demonstrated that HME-MUL-F2 exhibits antibacterial activity against pathogenic microorganisms such as *E. coli*, *S. aureus*, and *E. faecalis*, and promotes the proliferation of probiotics such as *L. rhamnosus* and *P. pentosaceus*. These findings were established in our previous study and were referenced in this study to provide context for the formulation’s potential effects on gut microbiota [28]. Similar outcomes have been reported in studies where anthocyanin-rich formulations positively influenced the gut microbial balance, promoting the proliferation of beneficial microbes [103].

Furthermore, in a Caco-2 and RAW 264.7 co-culture model, HME-MUL-F2 contributed to maintaining intestinal barrier integrity by increasing TEER levels, downregulating pro-inflammatory cytokines (*PGE2*, *IL-1β*, *TNF-α*, *IL-6*), and enhancing the expression of tight junction proteins (*ZO-1*, *occludin*, *JAM-1*, *claudin-1*, *-3*, *-4*), suggesting its potential role in mitigating inflammation-induced epithelial dysfunction. This finding is consistent with those of previous studies demonstrating that anthocyanins can enhance intestinal barrier function by modulating tight junction protein expression [104].

In vivo studies using a DSS-induced colitis mouse model further demonstrated that HME-MUL-F2 could alleviate colitis symptoms, increase body weight and colon length, reduce pro-inflammatory cytokines (*IL-1β*, *TNF-α*, *IL-6*, *MCP-1*), and increase anti-inflammatory cytokines (*IL-10*). This aligns with the results of existing literature highlighting the role of anthocyanins in attenuating inflammation by downregulating pro-inflammatory mediators [105]. Additionally, HME-MUL-F2 appeared to modulate the NF-κB signaling pathway and support gut microbial homeostasis, exhibiting comparable efficacy at a lower dosage (125 mg/kg) relative to that of MUL (250 mg/kg).

Our findings suggest that the observed therapeutic effects of HME-MUL-F2 may be primarily due to enhanced anthocyanin bioavailability and targeted delivery to the colon, leading to improved gut barrier function and modulation of inflammatory pathways, particularly through inhibition of the NF-κB signaling cascade.

These findings suggest that HME-MUL-F2 may serve as a promising formulation for enhancing gut barrier function and modulating the intestinal microbiome in IBD management. However, while improvements were observed in the treatment group, additional studies are required to determine whether these effects are directly attributable to increased anthocyanin delivery to the colon. Notably, its efficacy at a lower dose highlights its potential as an efficient therapeutic alternative. Furthermore, the application of HME technology to improve the stability and bioavailability of natural compounds underscores its broader prospects in regards to functional food development and targeted drug delivery systems.

Future research should focus on pharmacokinetic profiling, long-term safety assessments, and clinical validation to further substantiate the therapeutic potential of HME-MUL-F2. Continued investigations may facilitate its translation into functional foods, nutraceuticals, or natural therapeutics, while additional studies are warranted to comprehensively evaluate its therapeutic efficacy and clinical feasibility.

## Figures and Tables

**Table 1:** Anthocyanin contents of mulberry extract [28].

		Anthocyanin Content (mg/g DW)	
		C3G	C3R
Raw materials	MUL	43.13 ± 2.63 ^e^	2.99 ± 1.25 ^f^
Extrusion materials	HME-MUL	117.44 ± 1.44 ^d^	68.91 ± 1.35 ^e^
	HME-MUL-F2	325.02 ± 11.12 ^b^	154.73 ± 11.30 ^bc^

MUL—mulberry; MUL-HME—only solid formulations of the extrudate of MUL; HME-MUL-F2—treatment with mulberry HME-DDS formulation. Values having different letters (b–f) indicate significant differences obtained via Duncan’s multiple range test (DMRT, *p* < 0.05).

**Table 2:** Sequences of sense and antisense primers used for reverse transcription-quantitative PCR (RT-qPCR) of colon tissue.

Gene	Orientation	Primers Sequence (5′→3′)
*ZO-1* *(NM_175610)*	Forward	AAGGTTAGCTTACTGTCACACGCTT
	Reverse	ACCTAGAAGACATTGAAGGCATC
*Occludin* *(NM_002538)*	Forward	GTACCCACCAGTGACCAACA
	Reverse	GTTGCTGGAGCTTAGCCTGT
*JAM-1* *(NM_016946)*	Forward	ACAGCCATGAGGTCAGAGGCT
	Reverse	CGACTCTAGAAACACAAGAGCAAGA
*claudin-3* *(NM_001306)*	Forward	CACCACTACCAGCAGTCGATGAAC
	Reverse	AGACTGTGTGTCGTCTGTCACCATC
*claudin-4* *(NM_001305)*	Forward	GGTAGCTCAGCTGTGACTTTGGACTC
	Reverse	CTGGAGTAACGTGTAGGCTGAGTGAG
*GAPDH* *(NM_002046)*	Forward	TCCCACTCTTCCACCTTCGA
	Reverse	CAGGAAATGAGCTTGACAAAGTTG

## Data Availability

The original contributions presented in the study are included in the article, further inquiries can be directed to the corresponding author/s.
